# Supplementary material for: Differential Responses of Human iPSC-Derived Microglia to Stimulation with Diverse Inflammogens
Source: Cells. 2025 Oct 28;14(21):1687. doi: 10.3390/cells14211687 (PMC12607375; doi:10.3390/cells14211687)
Supplement: Supplementary file 1 [file cells-14-01687-s001.zip › cells-3923979-supplementary.pdf]

Supplementary information for

Differential responses of human iPSC-derived microglia to stimulation with diverse inflammogens

Chiara Wolfbeisz[1], Julian Suess[1], Nadine Dreser[1], Heidrun Leisner[1], Markus Brüll[1], Madeleine Fandrich[2], Nicole Schneiderhan-Marra[2], Oliver Poetz[2,3], Thomas Hartung[4,5,6], Marcel Leist[1,4]

[1]In vitro Toxicology and Biomedicine, Chair inaugurated by the Doerenkamp-Zbinden foundation, University of Konstanz, 78464 Konstanz, Germany
[2] Natural and Medical Sciences Institute, University of Tuebingen, 72074 Tuebingen, Germany
[3] SIGNATOPE GmbH, 72770 Reutlingen, Germany
[4] Center for Alternatives to Animal Testing (CAAT)-Europe, University of Konstanz, 78464 Konstanz, Germany
[5] Johns Hopkins University, Doerenkamp-Zbinden Chair for Evidence-based Toxicology, Baltimore, MD, USA
[6] CAAT, Johns Hopkins University, Baltimore, MD, USA
*Correspondence: marcel.leist@uni-konstanz.de; Tel.: +49-(0)-7531-88-5037

Table of Contents

**Fig. S1**	Applied gating strategy to quantify the marker expression of PreMacs	page 2
**Fig. S2**	Marker expression of PreMacs and MGLCs	page 3
**Fig. S3**	Comparison of gene expression between EBs, factories, PreMacs and MGLCs on DoC1 and DoC7	page 4
**Fig. S4**	MGLC morphology after stimulation with TLR ligands or cytokines	page 5
**Fig. S5**	Immunostainings of characteristic microglia markers	page 6
**Fig. S6**	Quality assessment of transcriptomic data	page 7
**Fig. S7**	Pattern of transcriptomic changes in microglia-like cells under diverse conditions	page 8
**Fig. S8**	Upregulated transcription factors identified by gene set enrichment analysis	page 9
**Fig. S9**	Translocation of NFκB in MGLCs after stimulation with TLR ligands or cytokines	page 10
**Fig. S10**	Phosphorylation of STAT1 and STAT3 in MGLCs after stimulation with TLR ligands or cytokines	page 11
**Fig. S11**	Translocation of NFκB in astrocytes after stimulation with TLR ligands or cytokines	page 12,13
**Tab. S1**	Antibodies used for immunofluorescence staining	page 14
**Tab. S2**	Antibodies used for Western blot	page 15
**Tab. S3**	Antibodies used for flow cytometry	page 16

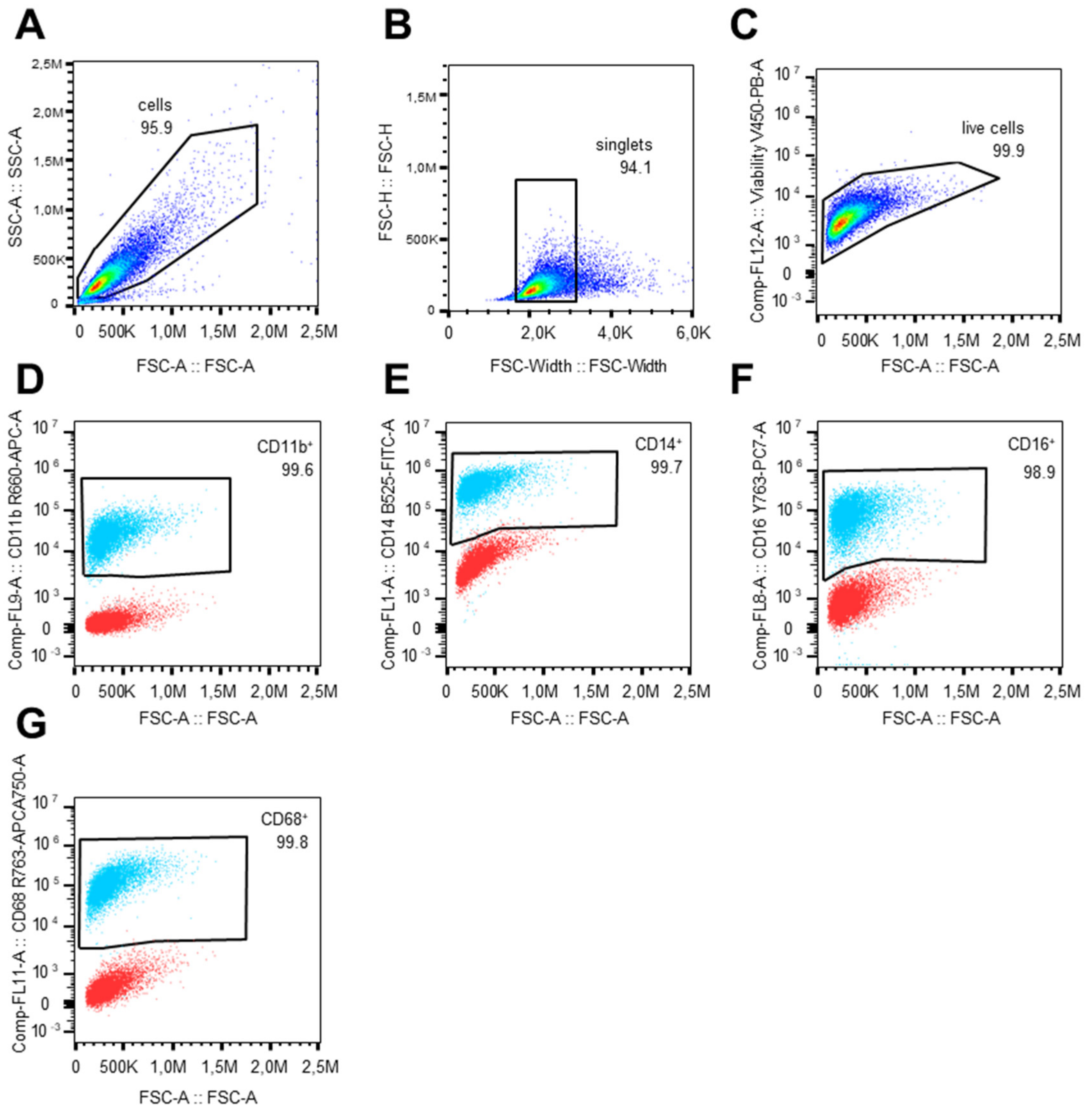

Fig. S1: Applied gating strategy to quantify the marker expression of PreMacs

PreMacs were stained and analyzed for the expression of the markers CD11b, CD14, CD16, and CD68 using flow cytometry directly after harvest from the factories. (A) Cells were separated from clumps and debris in the forward-sideward scatter area blot. (B) Doublet exclusion was performed by plotting the width of the forward scatter against its height. (C) A viability staining was used to distinguish live and dead cells. Live (viable) cells were negative for the stain, while non-viable (dead) cells were positively stained. (D) Viable cells were then individually gated for the markers (D) CD11b, (E) CD14, (F) CD16, and (G) CD68 (depicted in blue). Unstained cells functioned as negative controls (depicted in red).

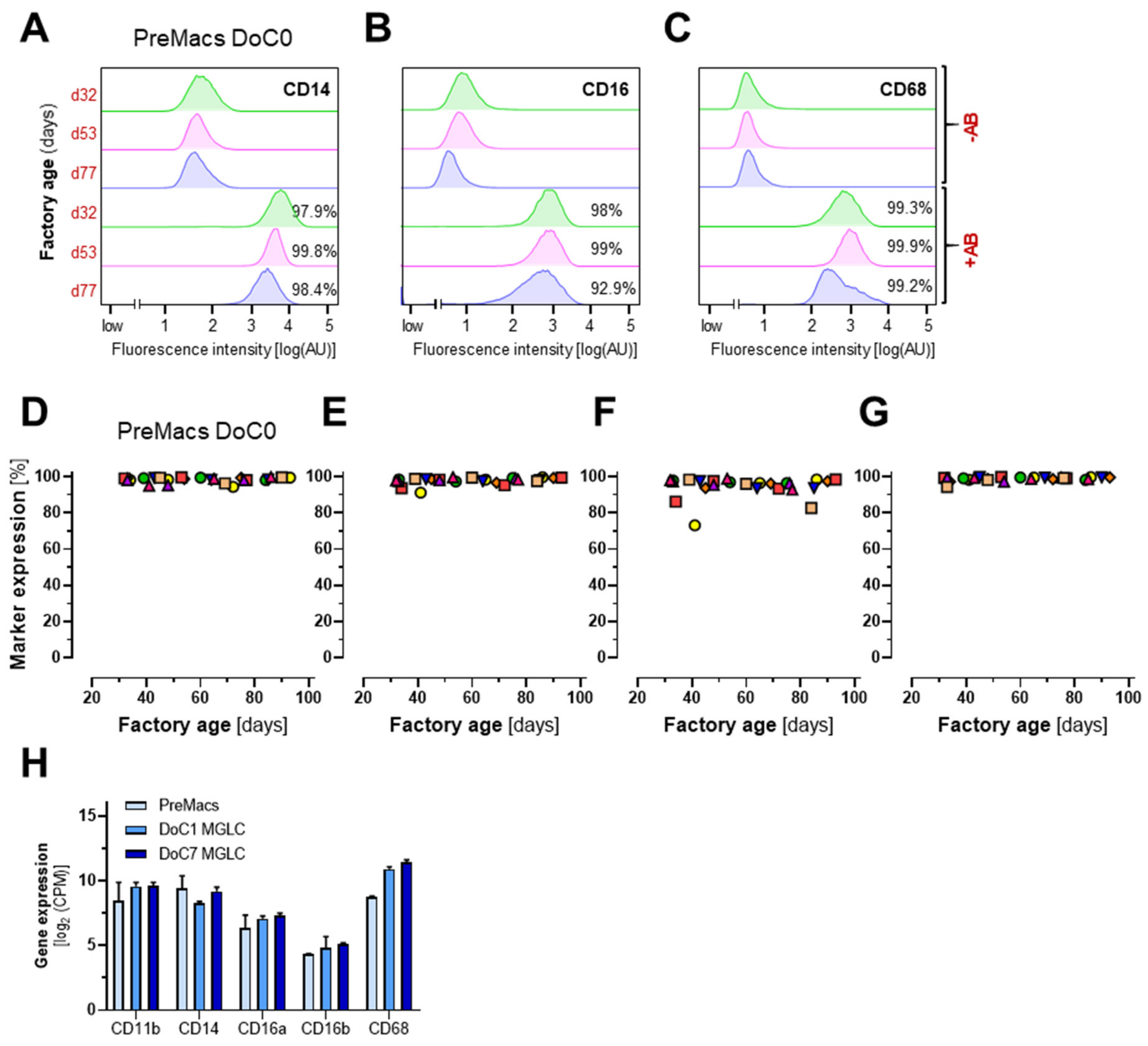

Fig. S2: Marker expression of PreMacs and MGLCs

Flow cytometry analysis of the markers CD14, CD16, and CD68 in PreMacs immediately after harvest from myeloid factories on days 32, 53, and 77. Cells were stained with (A) CD14, (B) CD16, and (C) CD68 antibodies (specific). The percentage of marker-positive cells is shown. These measurements correspond to the CD11b data presented in Fig 1C. Flow cytometry analysis of (D) CD11b, (E) CD14, (F) CD16, and (G) CD68 expression in PreMacs collected from the first harvest from the myeloid factories (approximately day 30) and at regular intervals up to 100 days of culture (N = 7). (H) Gene expression of CD11b, CD14, CD16, and CD68 for PreMacs, MGLCs on DoC1, DoC4, DoC7, DoC14 was determined by whole transcriptome analysis. The raw counts were normalized to counts per million total counts (CPM) and log_2-transformed (N = 2).

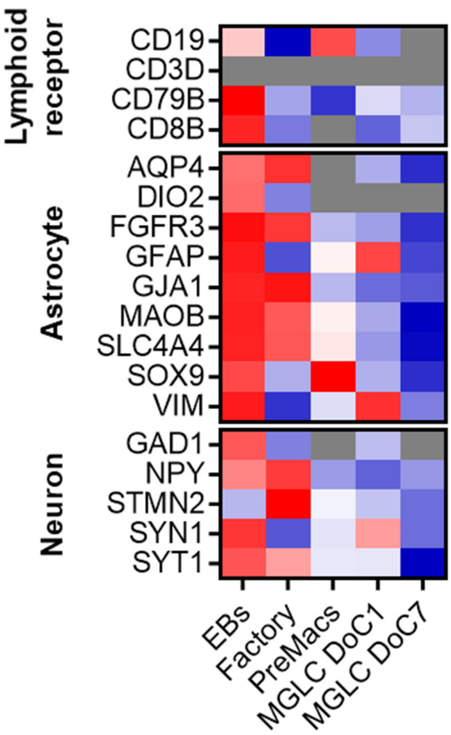

Fig. S3: Comparison of gene expression between EBs, factories, PreMacs and MGLCs on DoC1 and DoC7

Heat map depicting the row-wise z-scores of log_2 fold changes in EBs, factories (d45), PreMacs and MGLCs on DoC1 and DoC7 of selected marker genes for lymphoid cells, astrocytes and neurons. Full blue indicates a value of -1. Full red a value of +1. Less saturated colors indicate intermediate values. Zero being represented by pure white. Grey fields indicate samples with a signal below the detection limit (N = 2).

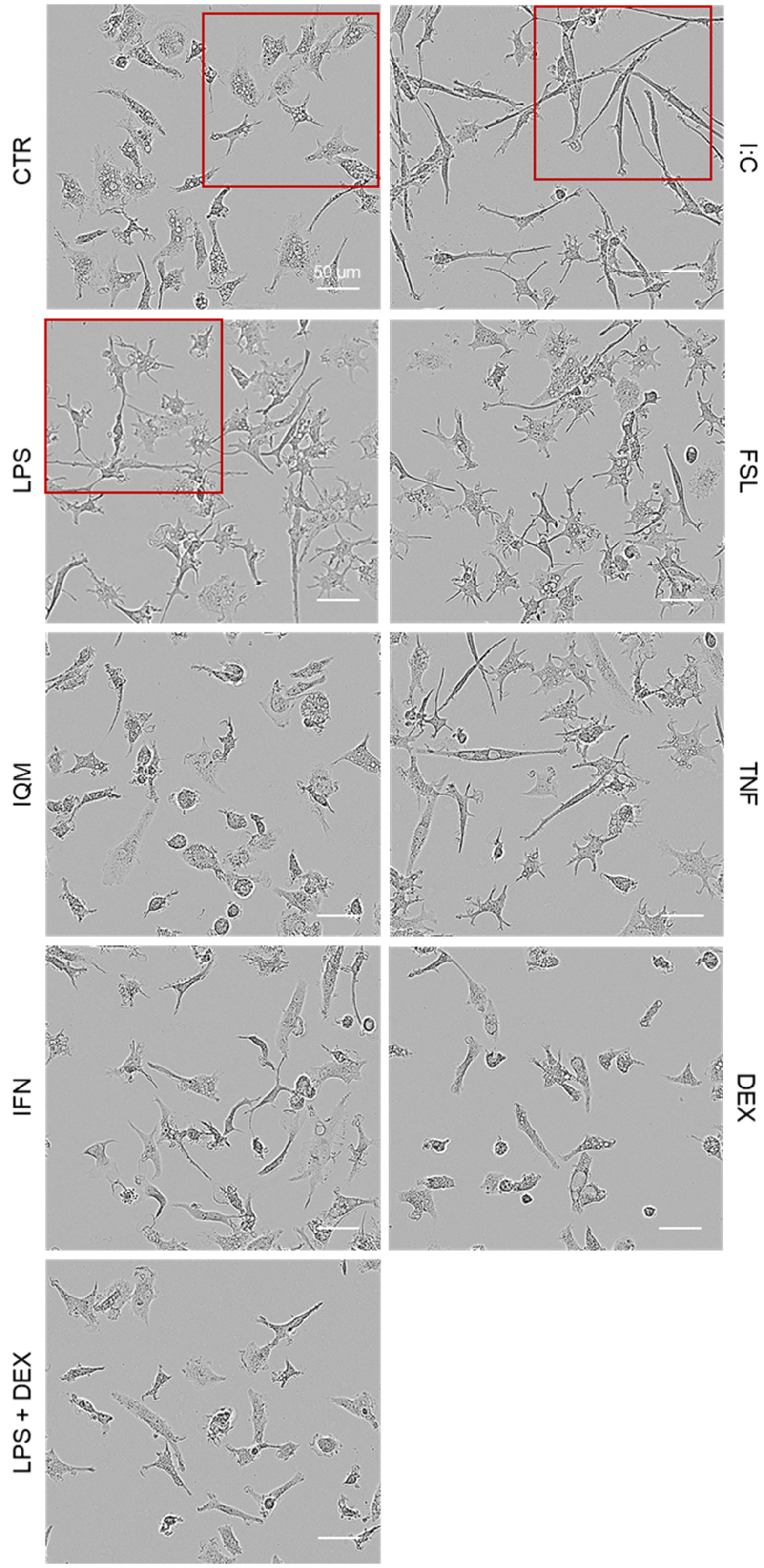

Fig. S4: MGLC morphology after stimulation with TLR ligands or cytokines

On DoC7, MGLCs were treated with different stimuli: LPS (100 ng/ml), I:C (10 µg/ml), FSL (100 ng/ml), IQM (10 µg/ml), TNF (10 ng/ml), IFNγ (20 ng/ml), DEX or LPS (100 ng/ml) *plus* DEX. After 48 h of treatment, MGLCs were imaged. Exemplary images are shown for all stimulations performed. The red boxes indicate the regions shown in Fig 1H.

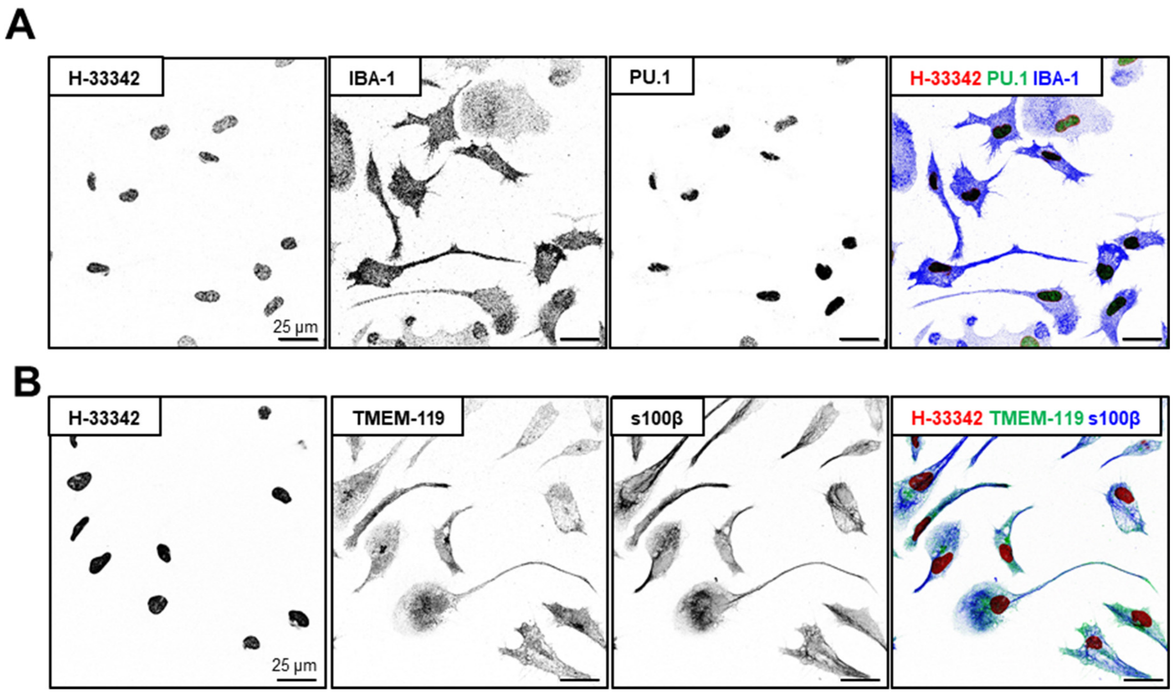

Fig. S5: Immunostainings of characteristic microglia markers

DoC7 MGLCs were fixed and immunostained for the following markers:

(A) IBA-1 (blue), PU.1 (green)

(B) TMEM119 (green), s100β (blue)

Nuclei were stained with H-33342 (red). Representative epifluorescence images of typical expression patterns and morphologies are shown.

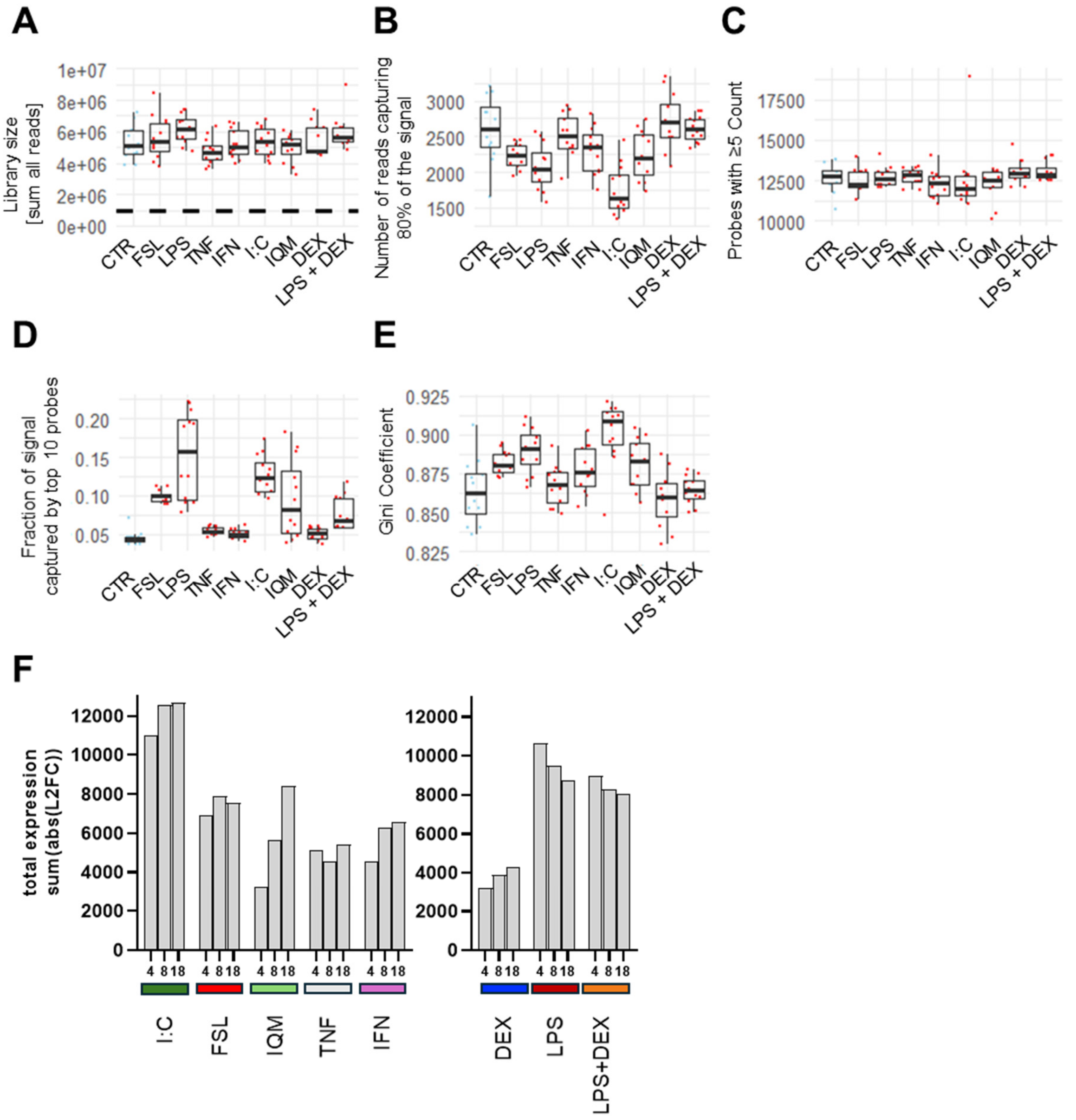

Fig. S6: Quality assessment of transcriptomic data

On DoD7, MGLCs were treated with different stimuli: LPS (100 ng/ml), I:C (10 µg/ml), FSL (100 ng/ml), IQM (10 µg/ml), TNF (10 ng/ml), IFN (20 ng/ml), DEX (100 µM) or LPS (100 ng/ml) *plus* DEX (100 µM). After the respective treatment period (4 h, 8 h, 18 h), cells were lysed and gene expression profiling was performed using TempOSeq analysis.

Quality assessment of transcriptomic data. A-E, Distributions of all sample-level quality control (QC) metrics, split by treatment. Dashed lines indicate thresholds for masking samples from further analysis.

(A) Library size, (B) number of reads capturing 80% of the signal, (C) probes with ≥ 5 count, (D) fraction of signal captured by top 10 probes, (E) Gini Coefficient.

(F) The sum of the log fold changes of all differentially expressed genes (DEGs) per stimulus and time point are depicted. Genes were considered as differentially expressed when the absolute $\log_2$ fold change was ≥ 1 and the adjusted p-value (padj) < 0.01 (N = 2).

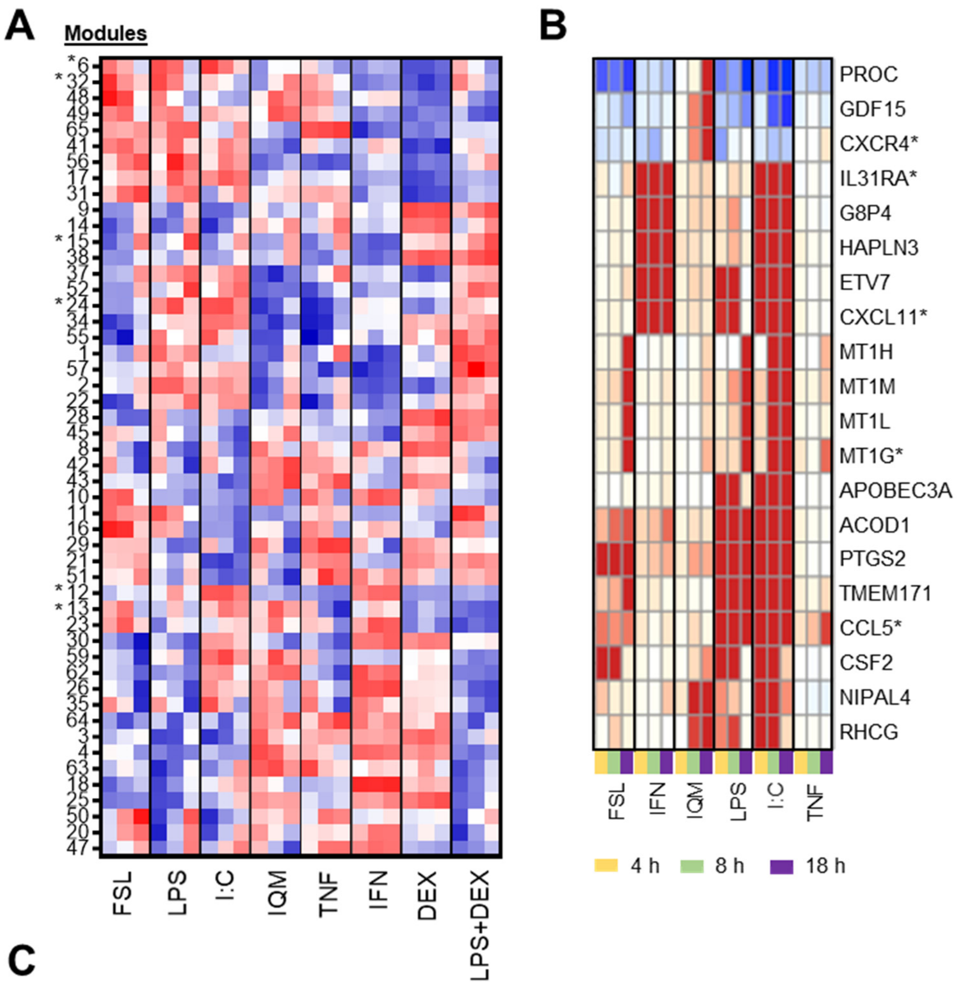

C

Gene	Comments / Functions
CXCR4	Receptor for CXCL12; directs immune cell migration in brain inflammation
IL31RA	Receptor for IL-31; receptor subunit or part of a receptor complex with oncostatin M receptor; pro-inflammatory
CXCL11	Also called IP-9 (Interferon-gamma-inducible protein 9); Ligand for CXCR3, CXCR7; induced by IFNγ, chemotactic for activated T cells
MT1G	Metallothionein; anti-oxidant defense (Nrf2-target); anti-inflammatory
CCL5	Also called RANTESLigand for CCR1, CCR3, CCR5; rather later released during inflammation

Fig. S7: Pattern of transcriptomic changes in microglia-like cells under diverse conditions

Changes in gene expression of MGLCs using different immunogenic stimuli relative to the untreated control. Co-induced genes are grouped into modules using Weighted Gene Co-expression Network Analysis (WGCNA) modules. Displayed are z-scores of the module eigengenes. A selection of all identified modules (see Fig S7A) is shown. The colors in the heatmap represent strength and direction of the correlation (red for positive and blue for negative correlations). The asterisk indicate the selection of modules shown in Figure 2F, G (N = 4). (B) Heatmap based on log_2 fold change values of genes selected from the top five absolute loadings of each of the first four principal components performed on log_2 fold change values“) (blue ≥ 3-fold down regulation; full red = ≥ 3-fold upregulation). Genes labelled by an asteriks show some exemplary patterns are explained below (N = 4). (C) Short background on exemplary genes from (B) (indicated by an asterik).

A

Highest — TF activation score — Lowest

4 h

LPS	NFKB1	RELA	NFKB	REL	JUN	STAT1	AP1	STAT3	IRF1
I:C	NFKB1	RELA	NFKB	STAT1	REL	IRF1	JUN	AP1	STAT3
FSL	NFKB1	RELA	NFKB	REL	JUN	NFKBIZ	HSF1	RELB	AP1
IQM	NFKB1	RELA	NFKB	REL	RELB	STAT3	AP1	CREB1	JUN
TNF	NFKB1	RELA	NFKB	REL	JUN	RELB	AP1	NFKB2	RBPJ
IFN	STAT1	IRF1	IRF2	IRF9	STAT2	NFKB1	RELA	DOT1L	NFKB
DEX	NR3C1	SALL1	FOXO3	CLOCK	BMAL2	NPAS2	SIRT1	HESX1	FOXO1
DEX + LPS	NFKB1	RELA	NFKB	REL	JUN	STAT3	RBPJ	HSF1	AP1

B

8 h

LPS	NFKB1	RELA	NFKB	REL	STAT1	JUN	IRF1	STAT3	AP1
I:C	NFKB1	RELA	STAT1	NFKB	IRF1	REL	JUN	STAT3	AP1
FSL	NFKB1	RELA	NFKB	REL	JUN	HSF1	RELB	STAT3	AP1
IQM	RELA	NFKB1	NFKB	JUN	AP1	REL	CREB1	CEBPG	RELB
TNF	RELA	NFKB1	NFKB	REL	RELB	JUN	IRF1	CREB1	AP1
IFN	STAT1	IRF1	IRF9	IRF2	STAT2	RELA	CIITA	NFKB1	DOT1L
DEX	NR3C1	FOXO3	FOXO1	CLOCK	SALL1	NPAS2	TBPL2	TRERF1	BHLHE40
DEX + LPS	NFKB1	RELA	NFKB	REL	STAT3	JUN	RBPJ	PITX3	AP1

C

18 h

LPS	NFKB1	RELA	NFKB	STAT3	REL	JUN	HIF1A	RBPJ	NFKBIB
I:C	NFKB1	STAT1	RELA	IRF1	NFKB	REL	JUN	STAT2	STAT3
FSL	NFKB1	RELA	NFKB	REL	STAT3	JUN	NFKBIZ	HSF1	NFKBIB
IQM	RELA	NFKB1	NFKB	AP1	JUN	HIF1A	EGR1	REL	HSF1
TNF	NFKB1	RELA	NFKB	REL	JUN	RELB	IRF1	STAT3	NFKBIB
IFN	IRF1	STAT1	CIITA	IRF2	IRF9	RELA	RFXAP	RFXANK	STAT2
DEX	FOXO3	NR3C1	TRERF1	FOXO1	SALL1	TBX3	SIRT1	SPDEF	BHLHE41
DEX + LPS	NR3C1	REL	PITX3	NFKB1	NFKB	FOXO3	DMTF1	SALL1	STAT3

Fig. S8: Upregulated transcription factors identified by gene set enrichment analysis

On DoD7, MGLCs were treated with different stimuli: LPS (100 ng/ml), I:C (10 µg/ml), FSL (100 ng/ml), IQM (10 µg/ml), TNF (10 ng/ml), IFN (20 ng/ml), DEX (100 µM) or LPS (100 ng/ml) *plus* DEX (100 µM). After the respective treatment period (4 h, 8 h, 18 h), cells were lysed and gene expression profiling was performed using TempOSeq analysis. The top ten upregulated transcription factors (TFs) determined by gene set enrichment analysis are displayed. TFs are arranged from left to right in order of decreasing activation score, with the highest activation scores on the left and the lowest on the right. (A) 4h, (B) 8h, (C) 18 h (N = 4).

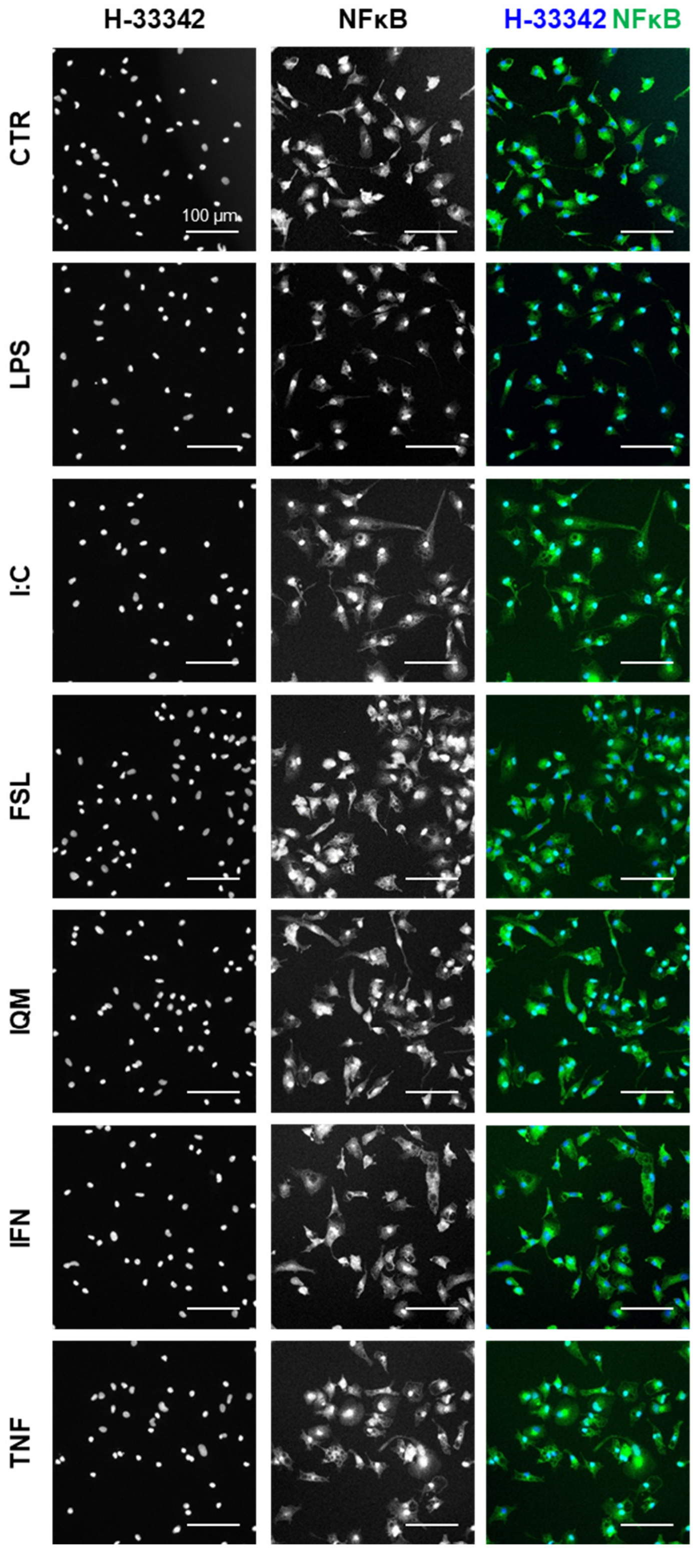

Fig. S9: Translocation of NFκB in MGLCs after stimulation with TLR ligands or cytokines

On DoC7, MGLCs were treated with different stimuli: LPS (100 ng/ml), I:C (10 µg/ml), FSL (100 ng/ml), IQM (10 µg/ml), TNF (10 ng/ml) or IFNγ (20 ng/ml). After 60 min of treatment, MGLCs were fixed and immunostained against the NFκB subunit p65. The nuclei were counterstained with Hoechst-33342. Exemplary images are shown which depict the same image fields as in Fig 3B, but give information on all fluorescent channels and their overlay.

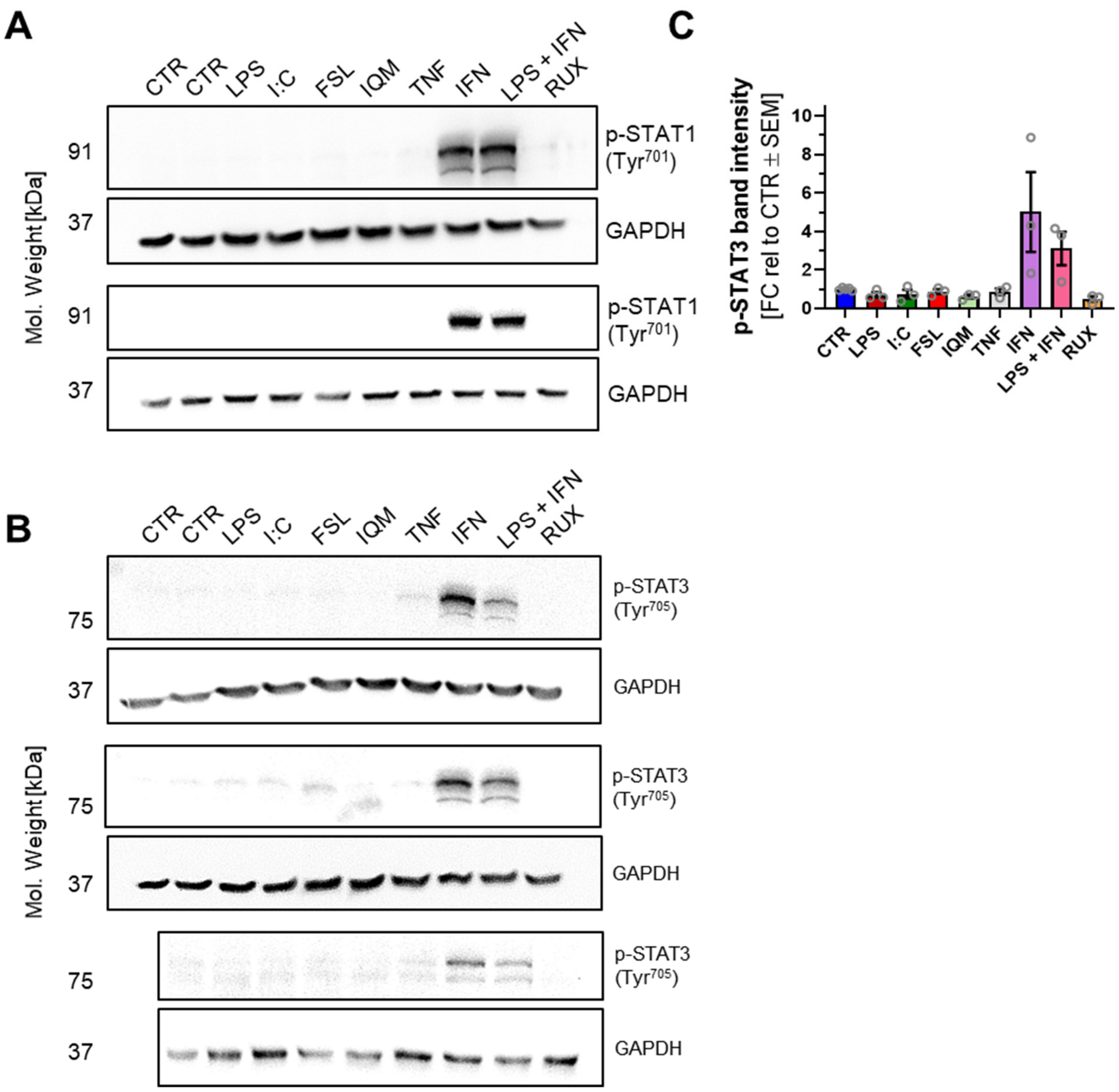

Fig. S10: Phosphorylation of STAT1 and STAT3 in MGLCs after stimulation with TLR ligands or cytokines

MGLCs were treated for 1 h with the following stimuli: LPS (100 ng/ml), I:C (10 µg/ml), FSL (100 ng/ml), IQM (10 µg/ml), TNF (10 ng/ml), IFN (20 ng/ml), LPS (100 ng/ml) *plus* IFNγ (20 ng/ml) and ruxolitinib (RUX, 20 µM) *plus* IFN (20 ng/ml) as combinatory treatment. For the combinatory treatment with RUX, MGLCs were preincubated for 30 min with Ruxo and subsequently treated with IFN for 60 min. Then, Western blot samples were taken. Western blots were performed with MGLC lysates, using (A) anti-phosphoSTAT1 (Tyr^{701}) or (B) anti-phosphoSTAT3 (Tyr^{705}) and anti-GAPDH antibodies used in (A) and (B). Three independent experiments were performed for phosphoSTAT1 (Tyr^{701}) (shown in Fig 3D and Fig S10A) and phosphoSTAT3 (Tyr^{705}) (shown in Supp Fig 10B) (N = 3). (C) Quantification of phosphoSTAT3 (Tyr^{705}) western blot bands. Bands were normalized to the intensity of the respective loading controls (GAPDH). Fold change is displayed relative to control. Data are from the three biological replicates shown in B (N = 3).

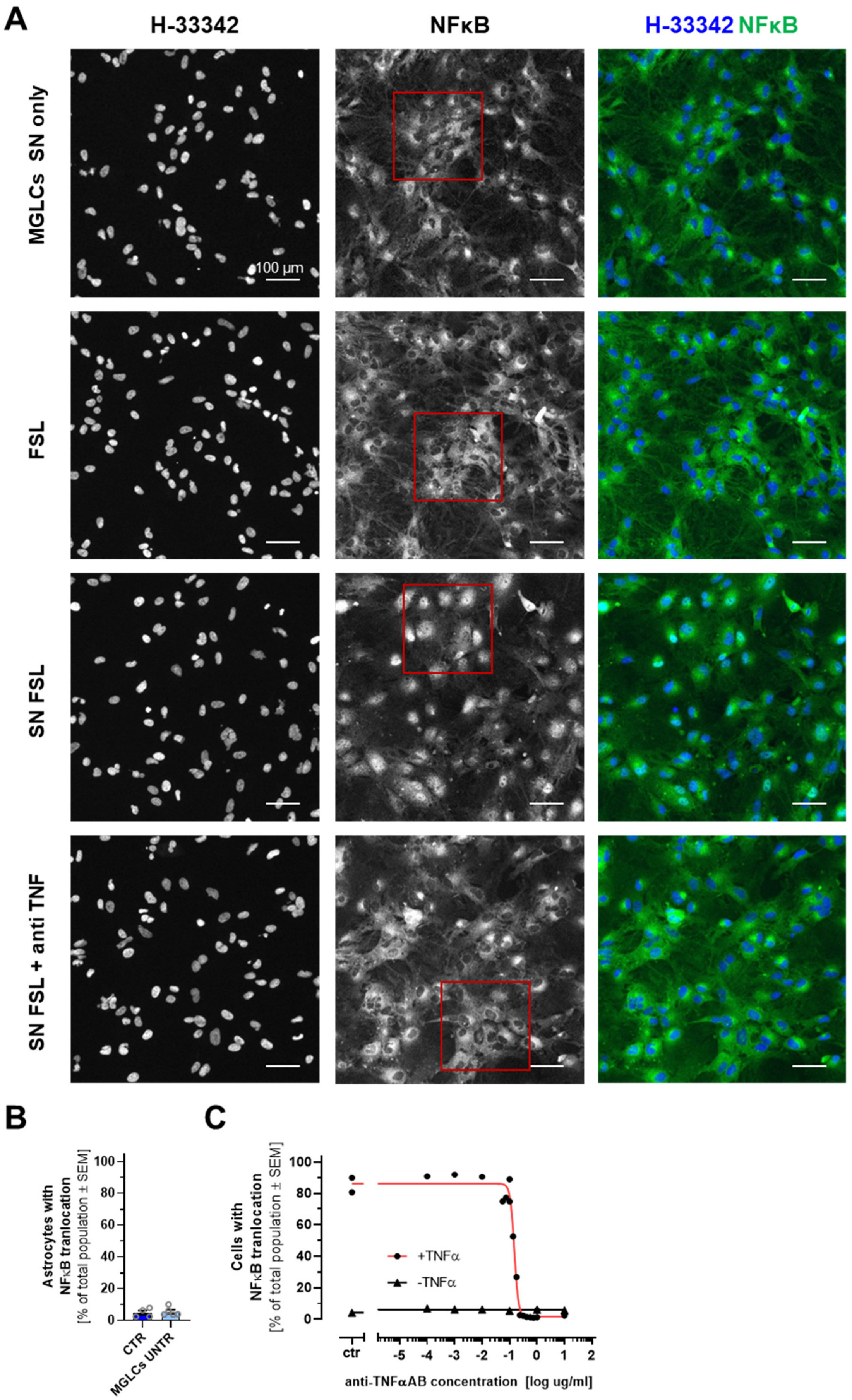
A
H-33342
NFκB
H-33342 NFκB
MGLCs SN only
FSL
SN FSL
SN FSL + anti TNF
100 µm
B
Astrocytes with NFκB tranlocation [% of total population ± SEM]
100
80
60
40
20
0
CTR
MGLCs UNTR
C
Cells with NFκB tranlocation [% of total population ± SEM]
+TNFα
-TNFα
ctr
-5
-4
-3
-2
-1
0
1
2
anti-TNFαAB concentration [log ug/ml]

Fig. S11: Translocation of NFκB in astrocytes after stimulation with TLR ligands or cytokines

(A) Astrocytes were treated with either the supernatant (SN) of unstimulated MGLCs, FSL (100 ng/ml), SN of MGLCs stimulated with FSL anti-TNF antibody. After 60 min of treatment, astrocytes were fixed and immunostained against the NFκB subunit p65. The nuclei were counterstained with Hoechst-33342. Exemplary images are shown which depict the same image fields (indicated by the red boxes) as in Fig 5C, F, G, J, but give information on all fluorescent channels and their overlay. (B) Medium either incubated for 18 h with or without MGLCs was transferred to astrocytes. After 60 min, NFκB translocation was quantified in astrocytes. These measurements correspond to the data presented in Fig 5E. Data are means of the biological replicates ± SEM (N = 4-5; n = 4). (C) For 2 h, TNF (20 ng/ml) was incubated with different anti-TNF antibody concentrations (highest: 10 ug/ml). This was transferred for 60 min onto astrocytes. Then, astrocytes were fixed and immunostained against the NFκB subunit p65. Data are shown as the percentage of cells with a nuclear NFκB p65 localization. The curve is the mean of the biological replicates which are respectively displayed by a circle/triangle each (N = 2; n = 4).

Table S1: Antibodies used for immunofluorescence staining

Target	Species	Dilution	Supplier	Catalogue number
**Primary**				
IBA-1	Goat	1:100	Abcam	AB5076
NFkB p65	Rabbit	1:200	BioLegend	901301
PU.1	Rabbit	1:300	Cell Signaling Technology	2258S
S100β	Mouse IgG1	1:100	Sigma	S 2532
TMEM-119	Rabbit	1:100	Sigma	HPA051870
**Secondary**				
Donkey anti-Goat IgG (H+L), Alexa Fluor 647		1:500	Invitrogen	**A-21447**
Donkey anti-Goat IgG (H+L), Alexa Fluor 488		1:500	Invitrogen	A-11055
Donkey anti-Rabbit IgG (H+L), Alexa Fluor 647		1:500	Invitrogen	A-31573
Goat anti-Mouse IgG1, Alexa Fluor 488		1:500	Invitrogen	A-21121
Goat anti-Mouse IgG1, Alexa Fluor 555		1:500	Invitrogen	A-21127
Goat anti-Rabbit IgG (H+L), Alexa Fluor 555		1:500	Invitrogen	A-21428

Table S2: Antibodies used for Western blot

Target	Species	Dilution	Supplier	Catalogue number
**Primary**				
P-STAT3 (Tyr705)	Rabbit	1:1000	Cell signaling	9145P
P-STAT1 (Tyr701)	Rabbit	1:1000	Cell signaling	9167
GAPDH	Mouse	1:5000	Abcam	Ab8245
**Secondary**				
Mouse	Goat anti-mouse HRP antibody	1:10000	Jackson Immuno Research	115-035-174
Rabbit	Donkey anti-rabbit HRP antibody	1:1000	Cell signaling	7074P2

Table S3: Antibodies used for flow cytometry

Target	Fluorochrome	Dilution	Supplier	Catalogue number
CD11	APC	1:50	Miltenyi Biotech	30-110-612
CD14	FITC	1:50	Miltenyi Biotech	130-110-576
CD16	PE-Vio 770	1:50	Miltenyi Biotech	130-113-956
CD68	APC-Vio 770	1:50	Miltenyi Biotech	130-114-654
Live-dead discriminator	Viobility 405/452 Fixable Dye	1:50	Miltenyi Biotech	115-035-174
